# Diverging Effects of Landscape Factors and Inter-Row Management on the Abundance of Beneficial and Herbivorous Arthropods in Andalusian Vineyards (Spain)

**DOI:** 10.3390/insects10100320

**Published:** 2019-09-26

**Authors:** Christine Judt, Gema Guzmán, José A. Gómez, José M. Cabezas, José A. Entrenas, Silvia Winter, Johann G. Zaller, Daniel Paredes

**Affiliations:** 1 Institute of Zoology, University of Natural Resources and Life Sciences Vienna (BOKU), 1180 Vienna, Austria; christine_judt@yahoo.com; 2 Institute for Sustainable Agriculture-CSIC, 14004 Córdoba, Spain; g92gudim@uco.es (G.G.); joseagomez@ias.csic.es (J.A.G.); josem.cabezas@juntadeandalucia.es (J.M.C.); p82enlej@uco.es (J.A.E.); 3 Institute of Integrative Nature Conservation Research, University of Natural Resources and Life Sciences Vienna (BOKU), 1180 Vienna, Austria; silvia.winter@boku.ac.at; 4 Institute of Plant Protection, University of Natural Resources and Life Sciences Vienna (BOKU), 1180 Vienna, Austria; 5 Departamento de Protección Ambiental, Grupo de Protección Vegetal, Estación Experimental de Zaidín, CSIC, 18008 Granada, Spain; daniel.paredes@eez.csic.es

**Keywords:** agroecosystems, arthropods, biological control, management effects, landscape ecology, viticulture, ecosystem services

## Abstract

Land use at landscape and field scales can increase the diversity and abundance of natural enemies for pest control. In this study, we investigated interactions between landscape elements (semi-natural vegetation, olive orchards, vineyards, other agricultural areas) and inter-row management (vegetation cover vs. bare soil) in relation to arthropod populations in Andalusian vineyards. Arthropods were collected from grapevine foliage in 15 vineyards using suction sampling. Landscape structure was analyzed within a 750 m radius surrounding the studied vineyards. Arthropods were categorized into functional groups (predators, parasitoids, herbivores), and their responses to the most influencing factors were analyzed by likelihood methods and model selection. Of the total of 650 arthropods collected, 48% were predators, 33% herbivores and 19% parasitoids. Numbers of predatory aeolothrips, parasitoids and herbivorous cicadas in the study vineyards decreased with an increased proportion of vineyards in the surroundings. Spider populations in vineyards increased with increasing proportions of other agricultural fields (non-flowering crops) in the surroundings. Semi-natural elements and olive orchards had no influence on the abundance of collected arthropods. We observed synergistic effects between landscape elements and inter-row management. The total numbers of arthropods, herbivores and parasitoids in vineyards benefitted from inter-row vegetation, while spiders benefitted from bare soil. Our findings underline the importance of both surrounding landscape elements and vineyard ground cover management to promote beneficial arthropods for potential natural pest control.

## 1. Introduction

The intensification of agriculture, with increasing field sizes at the expense of natural and semi-natural elements and high pesticide and fertilizer inputs, causes serious environmental problems including habitat and biodiversity loss [1,2,3]. However, biodiversity is strongly connected with ecosystem services such as natural pest regulation [4,5,6], and the conservation and protection of biological diversity has therefore become an important part of agri-environmental policies and science [7]. Moreover, conservation biological control (CBC) is increasingly seen as an alternative to the use of pesticides, especially in integrated production systems [8,9,10].

Practices stimulating CBC include the establishment, modification and management of natural and semi-natural elements (SNEs), such as cropland boundaries, hedgerows, fallows, grasslands, woodlands and forests. These SNEs can provide food, alternative prey and hosts, shelter, overwintering sites and other essential resources [11,12,13]. Sometimes, SNEs do not promote natural enemies [14,15] and can even foster disservices by providing habitats for pest species and crop diseases [16,17]. However, a number of studies have shown that the overall abundance and richness of natural enemies is generally higher in heterogenous landscapes with SNEs [18,19,20,21,22,23,24]. To increase natural pest control, it is also important to maintain diverse predator assemblages in agroecosystems [25,26,27], because of spatial and temporal differences in the diversity and abundance of generalist and specialist natural enemies [28,29]. A high proportion of SNEs surrounding crop fields can translate into pest suppression [24,30,31] but not always [23,32,33]. Also, improved local habitat quality, e.g., via vegetation cover, can enhance natural enemy populations and can be advantageous for crop production, especially in simple structured landscapes [2,9,26,34,35]. Ground cover with perennial crops commonly stimulates the abundance of beneficial arthropods [36,37,38,39,40] but this effect depends on various factors such as the type, composition and management of the cover crop; type and management of the field crop; type of natural enemies and pest arthropods; and climatic conditions [33,41,42,43]. However, most previous studies did not consider possible synergistic effects between ground cover and surrounding landscape structure on natural enemy populations in perennial crop fields [44,45].

The diversity of farming systems and the interactions of different types of habitats with natural enemies, as well as interactions between species, result in complex responses for different groups of arthropod natural enemies. Therefore, findings are often case-specific and difficult to translate to other agroecosystems [32,46]. Further, these interactions have mainly been studied in annual arable crop systems while less focus has been put on perennial crops, although pest regulation by natural enemies has been reported to be higher in the latter [47,48]. It seems that the generally lower level of disturbance and the permanency of both crop vegetation, such as grapevines, and non-crop vegetation do benefit natural enemies because arthropods can find alternative prey and/or shelter during periods of disturbance caused by crop management activities, e.g., the application of pesticides or tillage operations [49].

Vineyards are managed with different intensities and strategies. For example, the timing and frequency of herbicide applications or soil tillage of inter-row strips determines the diversity of the plants and arthropods that can inhabit these strips [50]. The integration of ecological and viticultural practices can produce win-win solutions for both wine growers and nature conservation [51]. Thus, a comprehensive understanding of how natural enemy populations are altered by SNEs in the surrounding landscape and vineyard inter-row management is necessary to foster natural pest control.

We hypothesized that in vineyards, inter-row vegetation cover and surrounding landscape elements influence arthropod populations on vines. The goal of this research was (i) to investigate the effects of inter-row management (bare soil vs. vegetation cover) on the abundance and diversity of arthropods on vines; (ii) to determine whether these effects are influenced by surrounding landscape elements and; (iii) to identify potential synergistic effects between inter-row management systems and surrounding landscape elements. Findings could enable different stakeholders to promote CBC while potentially reducing the use of insecticides and their negative effects on biodiversity and the environment.

## 2. Materials and Methods

### 2.1. Study Area

**Figure 1:**
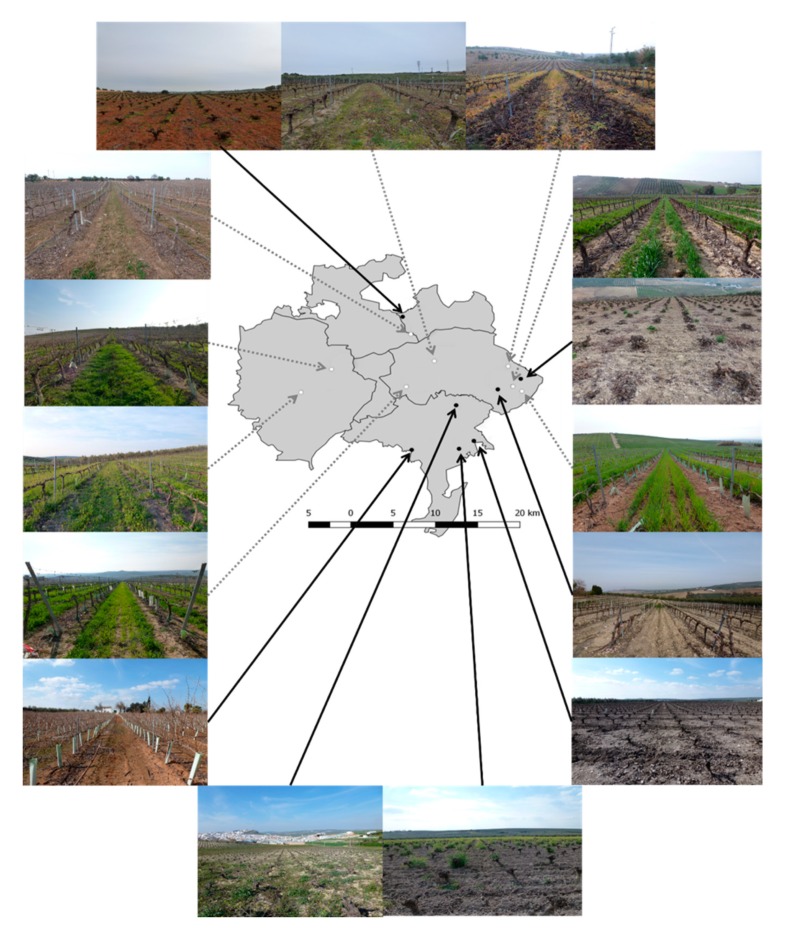
Views of the fifteen vineyards studied in the Montilla-Moriles wine region, near Córdoba, Andalusia, Spain. Black circles and solid arrows show vineyards with treatment bare soil; white circles and dotted arrows show treatment with inter-row vegetation cover. Adapted from [50].

The study was conducted in the Montilla-Moriles wine region, near Córdoba (37°38′–29′ N, 4°45′–31′ W), Andalusia, Spain. Although olive orchards dominate Andalusia’s agricultural landscape, winegrowing has a long tradition dating back at least to Roman times. In the study area, vines are cultivated on 5052 hectares at an altitude between 220 m and 682 m above sea level. Vineyards are interspersed with olive orchards, other agricultural crops, shrubs, grassland, grass stripes and tree rows (Figure 1). The region is characterized by a continental Mediterranean climate with an annual average temperature of 17.2 °C. Winters are mild and frosts are rare, while summers are typically hot with maximum daily temperatures of up to 40 °C. The hottest months are July and August with daily average temperatures of 28 °C. Grapes in this area receive between 2800 h and 3000 h of sunshine. The average annual rainfall is 604 mm, with precipitation mainly during the cooler winter months from December to February [52]. The typical soil types of the Montilla-Moriles region are “alberos” or “albarizas”, white soils that combine permeability with high moisture retention (around 30%) [53].

### 2.2. Experimental Design and Sampling

#### 2.2.1. Vineyards

Within the region, 15 conventional vineyards were selected according to differences in landscape complexity based on the proportion of semi-natural elements (SNEs) in the surrounding area. Vineyards were planted with the white grape variety Pedro Ximénez either in the traditional horizontal goblet system or in the trellis system (Figure 1). Within-row distances varied between 1.20 and 1.90 m, and inter-row distances between 1.75 m and 3.00 m. Fourteen vineyards were conventionally managed with similar fertilizer and pesticide inputs following recommended viticultural practices for the region, while one vineyard was cultivated according to the principles of organic farming.

Vineyards were classified into two groups regarding the inter-row management that had been implemented for at least the last three years. Eight vineyards had a temporary vegetation cover consisting of sown barley or a mixture including cereals, legumes and cruciferous plants, or had spontaneous vegetation (treatment vegetation cover). This vegetation was tilled or treated with herbicides at the beginning of March to prevent water competition. The other seven vineyards were more frequently tilled and/or treated with herbicides, resulting in bare soil throughout the year (treatment bare soil) [50].

#### 2.2.2. Arthropod Sampling

Collection of arthropods took place on 23 May 2016, when grapevine flowerhoods were separating (phenology state BBCH 57), and on 1 July 2016, when berries were pea-sized (BBCH 75). Our aim was to investigate arthropods that directly inhabit vines. Therefore, in each vineyard, samples of arthropods were taken from the inside of the foliage wall along one vine row per study vineyard. Sampling was conducted with a portable field aspirator (InsectaZooka, BioQuip Product, Inc. Rancho Dominguez, CA, USA) along 10 m of the vine foliage wall for 40 s. This was conducted six times, covering a transect of 60 m per vineyard. Vine rows in the region are commonly several hundred meters long. Thus, in total 180 samples were taken across the study vineyards. All captured arthropods were frozen before being identified using a light microscope and an identification key [54]. Details on inter-row vegetation in the study vineyards are given in [50]. Briefly, a total of 52 plant taxa were identified in the inter-rows across the vineyards. The number of different species identified at the inter-rows of the bare soil and cover crop vineyards was 32 and 44, respectively. The Sørensen index (IS) between the inter-rows of the bare soil and cover crop vineyards was 63.2%, indicating significant differences between the plant communities of the two inter-row treatments. Despite these differences in vegetation communities, some plant species such as *Brassica nigra* were present in all vineyards. 

### 2.3. Landscape Analysis

To assess the surrounding landscape structure, landscape elements within a 750 m radius around the center of the sampled vineyards were assessed by field mapping in 2016. Landscape elements were categorized according to CORINE Land Cover and EUNIS Habitat Classification into SNEs (hedges, tree rows, grass stripes, natural grassland, shrubs, woodlots, soft surfaced paths and roads and flowering crops), olive and other orchards, viticultural areas, other agricultural areas (non-flowering crops, mainly cereals), water items (ponds, rivers) and artificial/constructed entities (non-productive areas, urban areas, buildings and hard surfaced roads). Mapping and analysis were conducted using the programs ArcGis 10.2.1 [55], QGIS 2.8.1 [56], [57] FRAGSTATS 4.2 and CHLOE2012 [58].

### 2.4. Data Analysis

Due to the low number of individuals trapped, samples from both collection dates were pooled for each vineyard, resulting in one measurement per arthropod taxon per vineyard. Missing data from one vineyard were treated as not available (NA). Further, we examined data as one group (total) including all arthropod taxa and three subgroups summarizing predators, parasitoids and herbivores. As predators, we considered spiders (Araneae), aeolothrips (Thysanoptera: Aeolothripidae), ants (Hymenoptera: Formicidae), larvae of *Chrysoperla carnea* (Neuroptera: Chrysopidae), other net-winged insects (Neuroptera), flower bugs (Hemiptera: Anthocoridae), ladybirds (Coleoptera: Coccinellidae) and snakeflies (Raphidioptera). Insect parasitoids (Hymenoptera) were treated separately. As herbivores, we considered thrips (Thysanoptera), cicadas (Hemiptera: Cicadoidea), aphids (Hemiptera: Aphidoidea), psyllids (Hemiptera: Psyllidae) and grasshoppers (Orthoptera).

For data analysis, we used likelihood methods and model selection as an alternative to methods of traditional hypothesis testing [59]. We pre-selected explanatory variables according to their significance and frequency of occurrence. Then, we used Pearson correlations to check co-linearity among our selected explanatory variables (SNEs, vineyards, orchards, other agricultural areas). We established 13 linear models (LMs) using a Gaussian error distribution. Before computing the models, we log-transformed the response variables to better meet the assumption of this kind of analysis. We built four basic models, which contained only one of our explanatory variables (orchard area in percentage, vineyard area in percentage, other agricultural areas in percentage, area of semi-natural elements in percentage). The management models contained a combination of our explanatory variables and inter-row management, or the interaction of the landscape variables with inter-row management. We tested our models for arthropod groups (total arthropods, predators, herbivores, parasitoids) and for all arthropod groups, separately, that contained at least 25 individuals. Thus, we tested 143 models in total.

For model selection, we used the Akaike Information Criterion corrected for small sample sizes (AICc). The model with the lowest AICc and a difference of more than two units to the next AICc score was chosen as the “best” model. If multiple models did not fit the criteria and more than one model was plausible and contained the variable of the other model, we used the most complex one. The analyses were performed in R Studio [60] with R packages lme4 [61] and MuMIn [62].

## 3. Results

Landscape data analysis showed that the selected study sites were mainly surrounded by orchards (on average, 50% of the area), followed by vineyards (25%), other agricultural areas (10%) and SNEs (8%) (Table 1).

In total, 650 arthropod specimens were trapped on both data collection dates (Table 2). Of these, 314 individuals (ind., 48.3%) were predators, 120 ind. parasitoids (18.5%) and 216 ind. (33.2%) herbivores. Table 3 presents a comparison of alternative models representing those that best fit the different response variables. 

The resulting estimated parameters are shown in Table 4. More detailed results for our established groups (total and herbivores) and each relevant single group (parasitoids, spiders, aeolothrips, cicada) are given below. No best model could be identified for ants, aphids, thrips and grasshoppers. Total counts of larvae of *Chrysoperla carnea* and other net-winged insects, flower bugs, ladybirds, psyllids and snakeflies were too low to be further analyzed (Table 2).

**Figure 2:**
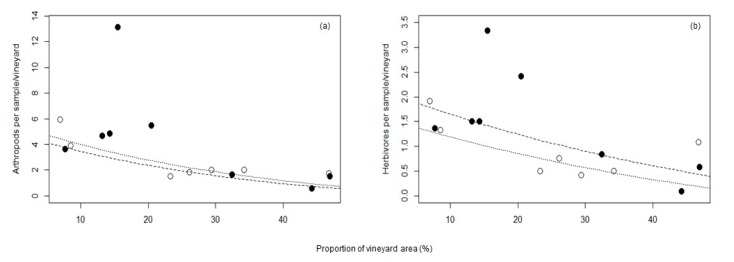
Model predictions and influence of landscape factors on the mean abundance of: (**a**) total arthropods; (**b**) the herbivore group. Dashed lines/black symbols represent the abundance of individuals in vineyards with vegetation cover, dotted lines/white symbols in vineyards with bare soil.

For total arthropods and the herbivore group, models including surrounding vineyard area and management showed the best fit (Table 3). In both groups, surrounding vineyard area had a negative effect on arthropod numbers in the studied vineyards. Thus, with increasing surrounding vineyard area, the mean abundance of total arthropods and herbivores decreased, but was generally higher in plots with vegetation cover compared to plots with bare soil (Figure 2).

In the case of our predator group, the best-fit models included agricultural area and vineyard area (Table 3). Because of two influencing independent variables (vineyard area, agricultural area), this model is no longer considered.

**Figure 3:**
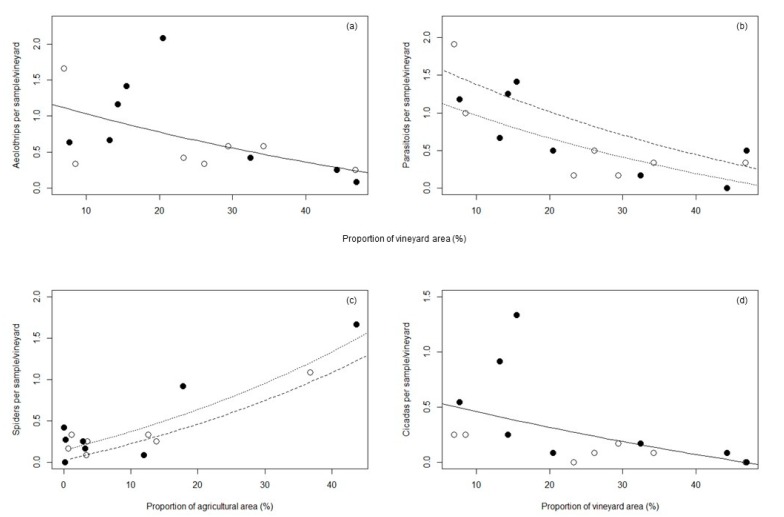
Model predictions and influence of landscape factors on the mean abundance of beneficial arthropods: (**a**) aeolothrips; (**b**) parasitoids; (**c**) spiders; and herbivorous insects: (**d**) cicadas. Dashed lines/black symbols represent the abundance of arthropods in vineyards with vegetation cover, dotted lines/white symbols in vineyards with bare soil. For aeolothrips and cicadas, no differences between vegetation cover and bare soil could be detected (black solid line).

Aeolothrips (130 ind. caught) were the most abundant beneficial insects (Table 2). Our best-fit model included only vineyard area (Table 3); abundance of aeolothrips decreased with increasing vineyard area (Figure 3a). Spider abundance (75 ind.) was best fit with a model containing the variables of agricultural area and inter-row management (Table 3). This was the only natural enemy group where we detected a positive influence of the surrounding landscape structure (agricultural area) on mean abundance, being higher in vineyards with bare soil than in those with vegetation cover (Figure 3c). 

Parasitoids (120 ind.) were the second most abundant natural enemy group (Table 2). As for aeolothrips, models including vineyard area and inter-row management fitted best (Table 3), with increasing vineyard area having a negative effect on the abundance of parasitoids (Figure 3b). Again, the mean abundance of parasitoids was higher in plots with vegetation cover.

Cicadas (50 ind.) represented the second most abundant herbivore group in the vineyards (Table 2). No influence of inter-row management on mean abundance was detected. The best fit model for cicadas included surrounding vineyard area (Table 3), exerting a slightly negative effect (Figure 3d).

## 4. Discussion

The present study is among the first to investigate the influence of surrounding landscape elements and inter-row soil management on the abundance of arthropods in Andalusian vineyards. We found contrasting effects on beneficial arthropods and on herbivores. This is in line with other studies from more temperate regions showing that habitat management in the field and landscape elements play an important role in affecting arthropods of different functional guilds [11,63,64,65].

While non-crop habitats close to crops are regarded as a source of beneficial populations [34,66,67,68], the SNEs in our study’s vineyards had little influence on the abundance of arthropods that we collected. This contrasts with some previous studies [28,44,69,70,71]. However, most previous studies examined annual arable crops, which differ from perennial crops such as grapevines in terms of frequencies of disturbance for sowing, soil cultivation and harvesting, the use of agrochemicals and resource availability over time [72,73,74,75]. It could also be that in our study the conditions in some elements of our SNE group (e.g., tree rows, grass strips) were still more hospitable to the arthropods than the vineyards, so they did not migrate into vineyards [76,77]. Furthermore, our SNEs consisted of 55% soft-surfaced roads, which do not provide appropriate habitats for the investigated arthropods. Additionally, we did not observe an effect of the surrounding olive orchards, the predominant crop in the region, indicating that little arthropod migration takes place between these two perennial cropping systems [70,71]. However, more detailed studies would be necessary to further investigate this.

The surrounding vineyard area always had a negative impact on the abundance of arthropods in the studied vineyards, suggesting that arthropod populations might be diluted across vineyards in the landscape. A similar pattern has also been observed for oilseed rape [30]. Furthermore, the effect was most pronounced on aeolothrips, parasitoids and cicadas, suggesting different responses to disturbance and habitat characteristics as well as different dispersal ranges. [64,74,78,79]. In any case, our findings indicate the importance of heterogenous landscapes in order to sustain a broad diversity of natural enemies [80,81]. 

Further, we found that other agricultural areas surrounding vineyards (i.e., non-flowering crops such as cereals) increased the occurrence of spiders in the vineyards. First, this could mean that these areas may have functioned as a source for spiders, from where they migrated into the vineyards [70]. Second, and particularly in landscapes with arid conditions, perennial crops such as vineyards with vegetation cover could have provided better resources for spiders than other agricultural areas did. Indeed, we detected synergistic effects between landscape factors and inter-row management. At least for total arthropods, herbivores and parasitoids, vegetation cover had a positive influence [74,82]. In the case of parasitoids, the positive synergistic effect between surrounding vineyard area and vegetation cover within the sampled vineyards might also be due to the host- and habitat-specification of parasitoids [64,83] and thus to their stronger response to landscape complexity at smaller scales [28]. Our results on parasitoids underline previous findings that vegetation cover could be beneficial for natural enemies [38,84,85,86] by providing resources such as nectar, pollen, alternative hosts and shelter [40,70,87,88].

Spiders were more abundant in vineyards with bare soil. This is in contrast to an earlier finding that total spider densities in vineyards were unaffected by vegetation cover [89], and other studies reporting positive effects of vegetation cover on spider populations [33,37,90]. However, these contrasting effects are perhaps due to considering spiders in the vegetation cover versus spiders on the vines [33,89,91,92]. We assume that the higher spider abundance in plots with bare soil was due to a lack of prey on bare soil and a preference for the foliage wall [93,94]. Moreover, climatic preferences and especially humidity on the soil surface could have played an important role [29,33]. 

Also, climatic factors, e.g., July being one of the hottest and driest months in the year, could be the reason for the rather low arthropod abundances in our study.

## 5. Conclusions

In summary, our study is a first attempt to investigate inter-relationships between vineyard management and landscape structure on a variety of arthropod taxa in an infrequently studied Mediterranean vineyard ecosystem. The findings show that both landscape elements and field management practices affect the abundance of arthropods in vineyards. We found little influence of SNEs on vineyard arthropods, but a positive effect of vegetation cover in vineyards on some natural enemy taxa. However, the patterns found have to be interpreted with caution as the observed arthropod abundances were rather low. Assessments of predation and parasitism rates of the most important pest taxa (e.g., grape berry moths) would be necessary to gain a more comprehensive understanding of further effects on potential natural pest control. Based on these results, we recommend integrating local management and landscape structure parameters in existing pest management strategies. 

## Figures and Tables

**Table 1:** Surrounding landscape types for the studied vineyards. Numbers are mean percentages ± SD.

Landscape Structure	Inter-Row Management	
	Bare Soil (in %)	Vegetation Cover (in %)
Semi-natural elements ^1^	7.9 ± 3.3	9.0 ± 3.1
Orchards ^2^	50.8 ± 7.8	49.6 ± 17.2
Vineyards	26.5 ± 12.5	23.1 ± 15.9
Other agriculture ^3^	5.5 ± 6.9	14.1 ± 17.0

^1^ 55% consisted of soft-surfaced agricultural roads, 18% tree roads, 16% grass stripes, 4% hedges, 3% natural grassland, 1.5% shrubs and grassland, 1% woodlots, 0.2% flowering crops; ^2^ 98% olive orchards; ^3^ cereals.

**Table 2:** Overview of main arthropod groups (predators, parasitoids, herbivores, in bold letters) and respective taxa trapped in vineyard plots on both sampling dates.

Arthropod Taxa	Counts	% of Total Catch
**Predators**	**314**	**48.3**
Aeolothrips	130	20.0
Ants	97	14.9
Spiders	75	11.5
*Chrysoperla carnea larvae*	6	0.9
Cocinellidae	2	0.3
Neuroptera	2	0.3
Raphidioptera	1	0.15
Anthocorids	1	0.15
**Parasitoids**	**120**	**18.5**
**Herbivores**	**216**	**33.2**
Thrips	72	11.1
Cicada	50	7.7
Grasshoppers	48	7.3
Aphids	27	4.2
Psyllids	19	2.9
**Total arthropods**	**650**	**100**

**Table 3:** Comparison of alternative models (using Akaike Information Criterion corrected for small sample sizes, AICc) for the main arthropods and groups found in Andalusian vineyards. The best model(s) is indicated in bold font. SNE = semi-natural elements.

Model Parameter	Total	Predators	Herbivores	Aeolothrips	Parasitoids	Spiders	Cicadas
**Basic models**							
Null	29.0	26.0	16.6	11.0	11.9	7.3	4.6
SNE	32.2	29.1	19.8	14.1	15.1	9.6	7.6
Other agric.	26.3	**22.8**	15.8	13.7	9.8	**−10.3**	4.5
Viticulture	**20.2**	**21.5**	11.4	**7.1**	**2.1**	6.6	**1.5**
Orchards	31.3	28.6	18.9	10.4	14.9	9.5	7.4
**Management models**							
Null	29.0	27.9	14.4	13.7	12.4	10.4	7.1
SNE + management	32.7	31.7	17.8	17.5	16.2	13.1	10.6
SNE *×* management	37.1	35.5	22.3	22.1	20.1	15.8	15.2
Other agric. + management	28.4	26.5	15.8	17.3	12.3	**−9.2**	8.2
Other agric. *×* management	33.1	31.1	20.2	21.6	16.8	−4.6	11.8
Viticulture + management	**19.4**	24.3	**7.8**	10.7	**2.0**	10.4	4.9
Viticulture *×* management	24.0	27.8	11.6	15.1	4.3	15.0	9.4
Orchards + management	31.4	31.0	16.5	13.4	15.9	13.2	10.4
Orchards *×* management	35.7	35.7	20.8	17.9	19.4	17.8	14.5
Multiple R^2^	0.67	-	0.65	0.38	0.58	0.79	0.34
Adjusted R^2^	0.62	-	0.59	0.33	0.55	0.76	0.29

**Table 4:** Parameter estimates for the selected best models of the abundance of individual arthropod taxa and groups. Semi-natural elements and orchards were not among the selected models and are therefore not shown.

Taxa	Estimates			
	Intercept	Vineyard as % of Surrounding Area	Agric. Land as % of Surrounding Area	Presence of Vegetation Cover
Total arthropods	1.86	−0.028	-	0.38
Herbivores	0.93	−0.01	-	0.34
Aeolothrips	0.83	−0.02	-	-
Parasitoids	0.83	−0.02	-	0.19
Spiders	0.15	-	0.02	−0.11
Cicada	0.47	−0.01	-	-
